# Trade-Off Between Schooling and Labor for Children: Understanding the Determinative Factors Among Rural Households in Bangladesh

**DOI:** 10.3389/fsoc.2022.839231

**Published:** 2022-06-14

**Authors:** Rafiqul Islam, Md Mahmudul Hoque

**Affiliations:** Ministry of Public Administration, Dhaka, Bangladesh

**Keywords:** child labor, trade-off, schooling, factors, parents, rural households, Bangladesh

## Abstract

This research is concerned with understanding the factors behind the trade-off between child labor and child schooling, given the well-documented links between the two. It examines parents' behavior in their decision-making on their children's schooling or practicing child labor. Depending on qualitative research methods including 28 semi-structured interviews and two focus group discussions conducted in the rural areas of Bangladesh in 2020, this study reveals the following: subsistence needs compel households, particularly the ultra-poor and the female-headed, to trade off child labor with schooling; due to higher demand of labor, parents engage their children into work instead of schooling; parents of labor-intensive occupations tend to trade off child labor with schooling; sexual division of labor remains obvious; finally, credit constraints and cultural beliefs have negative impacts on parental decision-making on child schooling. Interventions aiming to reduce child labor and increase schooling in these rural areas must remain mindful of the socio-economic and cultural needs.

## Introduction

International Labor Organization's Convention No. 182 illustrates two kinds of adverse effects of child labor—one is the direct effect on the child's physical, mental, or social development and another is the indirect effect on the child's schooling (ILO, 1999). Several studies related to child labor also highlight its effect on schooling (Binder and Scrogin, 1999; Ravallion and Wodon, 2000; Ray, 2000; Parikh and Sadoulet, 2005). This research focuses on the trading-off between labor and schooling for children in a developing country context to further this understanding. Although the harmful effects of child labor are generally acknowledged by adults, parents in poor and developing economic contexts are often induced by its monetary returns. Parents often find it hard to bear the costs of schooling (Delap, 2001). Formal schooling is considered a vital contributor to human skills and capital (Mussida et al., 2019; Posso, 2020). Target 4.1 of the 2015 Global Development Agenda calls for ensuring that all girls and boys complete free, equitable, and quality primary and secondary education (Boeren, 2019). However, the growing number of school dropouts in developing countries remains a global concern (UNESCO, 2015; Hossain and Akter, 2019; Sarker et al., 2019). This qualitative investigation explores the household-level factors in the rural areas of Bangladesh to explain why parents trade off children's schooling with labor.

Previous studies have used various lenses to focus on the issue. Some works analyzed household demographic factors (Grootaert and Kanbur, 1995; Basu and Van, 1998; Salmon, 2005; Huisman and Smits, 2009), while others extensively focused on socio-cultural factors and the intra-household demand and supply of labor (Delap, 2001; Bhalotra and Heady, 2003; Emerson and Souza, 2003; Mukherjee and Pal, 2016; Hoque, 2021a). Several empirical findings indicate that parents engaged in labor-intensive occupations tend to trade off schooling with child labor in various localized contexts (Bhalotra and Heady, 2003; Haile and Haile, 2012; Yokying and Floro, 2020).

A few empirical studies have also examined the intra-household decision-making regarding children's work and labor in rural and urban areas in Bangladesh, and recognized parental occupation as a significant determinant (Amin et al., 2006; Shafiq, 2007; Tariquzzaman and Hossain, 2009). Drawing from the aforementioned works, this study contributes to the empirical literature by focusing on the parents' occupations in explaining their decisions regarding trade-offs between schooling and the labor of children in rural areas in Bangladesh. Assuming that parents who are engaged in labor-intensive occupations in low-income families in rural areas tend to engage their children in child labor, this study examines whether government interventions influence the respective parental decisions. To our knowledge, no qualitative empirical studies have purely focused on this issue. Based on this assumption, this research was led by two questions: (i) What are the factors that influence households' decisions in trading off between schooling and child labor in rural areas of Bangladesh? (ii) What government and non-government interventions are perceived to be effective? This study adopted qualitative primary research to address these questions. This paper is organized as follows. The next section outlines the review of literature followed by the research methodology. The subsequent section delineates the findings and analysis. The final section concludes with highlighting the key implications of this study.

## Literature Review

### Factors of Child Labor and Schooling

Child labor is generally considered harmful as it takes away a child's childhood and inhibits their social mobility. International Labor Organization (ILO) defines child labor as “work that is mentally, physically, socially or morally dangerous and harmful to children and interferes with their schooling” (ILO, 2002, p. 15). The latest global estimates state that about 160 million children aged 5–17−63 million girls and 97 million boys—were engaged in child labor globally in 2020 (ILO and UNICEF, 2021). Three major international conventions that set the standards for child employment/labor are—ILO Convention No. 138 on Minimum Age for Admission to Employment in 1973, ILO's Worst Forms of Child Labor Convention, 1999 (No. 182) and United Nations Convention on the Rights of the Child, 1989 (UNCRC). Following these conventions, child labor was seen as an unaccepable practice until the mid-1990s when emerging evidence started to back up a common understanding that not all work was harmful for children, and that employment in certain, safe kinds of work could be beneficial to achieve survival level of consumptions and skills (Rogers and Swinnerton, 2002). For instance, Mergos (1992) discovered that children had a positive economic contribution to farm households through peasant agriculture work in the Philipines. Bachman (2000) argues that stopping child labor may bring a halt to the survival incomes of many poor families. Recognizing schooling as a fundamental right and need of children, several studies have idenfiied child labor as a major obstacle in low-income family contexts (Ravallion and Wodon, 2000; Ray, 2000; Parikh and Sadoulet, 2005). Working hours was also found responsible for reducing children's leisure and outdoor activities (Binder and Scrogin, 1999).

Parental and household behavior regarding sending a child to school depends on a wide range of factors. Theoretical and empirical studies have interrogated the trade-off between schooling and labor mainly in low-income contexts. Basu and Van's (1998) luxary axiom states that a family engages in labor only when the imcome of adult family members is very low (Swinnerton and Rogers, 1999). Poverty was identified as a major driver of child labor. Jensen and Nielsen's (1997) study revealed that parents could not send their children to school in Zambia due to lack of monetary resources. Buchmann and Brakewood (2000) have drawn similar observations in Thailand and Kenya. Shafiq (2007) and Malik (2013) revealed the trade-off between school enrolment and child labor due to poverty in India and Bangladesh, respectively. However, evidence from various contexts also commonly indicates that the supply of child labor depends on the wage, demand, and opportunities in the market (Parikh and Sadoulet, 2005; Roy et al., 2015; Mukherjee and Pal, 2016; Tama et al., 2018).

Besides poverty, several other factors drive households to trade-off between schooling and child labor. Demographic factors like children's age, number of children and family members, and absence of a parent influence the decision (Tariquzzaman and Hossain, 2009; Malik, 2013; Khan and Lyon, 2015). Duraisamy's (2000) research based in India revealed that with age, children's participation in work increases, and schooling decreases. So, in other words, an older child is at more risk in trading-off schooling with child labor (Ray, 2000). In Bangladesh's rural areas, meanwhile, schooling of older boys depends on the number of children. If the number is high, the probability of schooling is low as the older siblings have to take care of the younger ones (Amin et al., 2006). However, several empirical researches conducted in Bangladesh showed that in many cases the older children often combine work and schooling (Alam et al., 2008; Khanam, 2008; Quattri and Watkins, 2019). It is well-established in the literature that when schools are close to where the children live, this may increase study hours and school attendance (Jensen and Nielsen, 1997; Huisman and Smits, 2009). Ray (2000) found that infrastructure such as the availability of classrooms can reduce child labor hours by increasing school hours.

Social and cultural factors are also critical. Delap (2001) argues that besides the economic factors, child labor crucially involves societal norms related to gender and culture. Children's sex, age, and place of residence appear to play important roles in determining the type of work they perform in communities across rural and urban Bangladesh (Delap, 2000; Salmon, 2005; Tariquzzaman and Hossain, 2009; UCW, 2011; Hoque, 2021b). Perhaps interestingly, there are still some dichotomies in the literature. For example, Khanam (2005, 2008) observed that girls in rural areas must combine household work with schooling to a much greater extent than boys in Bangladesh's rural areas, while in contrast, Shafiq (2007) found no discrimination based on gender in rural households in Bangladesh.

### Parents' Occupation and Education

Parental characteristics (i.e., occupation, education, type of employment, and so forth) play an important role in deciding how their children will spend their time. Evidence suggests that most children are employed by their parents for domestic help or to work in family farms and businesses (Fors, 2012; ILO UNICEF, 2021). Parikh and Sadoulet (2005) found in Brazil that self-employed or employer parents engage their children into labor more than employee parents. This indicates that availability or opportunities of work is a determinant of child labor. In Bangladesh, parents in non-labor-intensive occupations generally do not send their children to work, and wealthier rural households in Bangladesh can keep their children in school by bearing the opportunity cost (Amin et al., 2006). However, Bhalotra and Heady (2003) explained how the opposite could also happen. They called this the “wealth-paradox” phenomenon, which is that asset-rich households may engage their children into labor more than the asset-poor households. Due to credit constraints, asset-rich households may not be able to hire labor for their farm, so they may deploy their children into their farms. Several empirical research findings support this paradox (Buchmann and Brakewood, 2000; Ravallion and Wodon, 2000; Baschieri and Falkingham, 2009). Rammohan (2012) found in India that households that own agricultural lands usually let their children combine schooling and work; still, they may pull the children out of school during the harvest season when the demand for labor is higher. Salmon (2005) states that children are generally employed as unpaid labor in the agricultural sector, which indicates the high demand for child labor in labor-intensive occupations. Similarly, Rahman et al. (2010) found that children in rural Bangladesh are more likely to work in their parents' occupations in the agricultural sector.

In poor communities, households or families with disabled or illiterate parents are more likely to opt for generating income by engaging their children in child labor (Edmonds, 2007; Webbink et al., 2012). Several studies show that parents' education has a positive impact on child schooling and negative impact on child labor in Bangladesh (Ravallion and Wodon, 2000; Khanam, 2005; Shafiq, 2007; Ahmed and Ray, 2011; Hossain and Akter, 2019). Ahmed and Ray (2011) reveal that parents' level of education affects the trade-off decision-making across genders; however, a mother's education shows a significant inclination toward educating a girl child. Based on data collected from two districts of Bangladesh Hossain and Akter (2019) observe that children of educated parents left school less than less-educated parents.

### Determinants of Parents' Decision-Making on Schooling or Child Labor

The return from investment of human capital is greater than the return from investing in physical assets (Schultz, 1989). However, when it comes to decision-making, parents often favor having their children combining child labor with informal learning (e.g., home education, religious studies, NGO-run non-formal education). Parental beliefs about the returns from schooling in forming human capital such as skills, knowledge, and health remain a primary determinant (Mukherjee and Pal, 2016). Many parents believe formal education fails to ensure social mobility for their children (Bazin and Bhukuth, 2009). While analyzing urban child labor among poor households in Bangladesh, Tariquzzaman and Hossain (2009) identified parental perception of the low returns from formal education as a significant influence. Poor quality educational provision, low attainment and high dropout rates among poor children are the key factors behind this perception (Tariquzzaman and Hossain, 2009). Some other recognized critical determinants are the distance of schools, low return on human capital investment, large indirect cost of schooling, and low quality of educational facilities (Jensen and Nielsen, 1997). Huisman and Smits (2009) studied 220,000 children in 340 districts of 30 developing countries to find out household and district level determinants of primary school enrolment and found that parental decisions on children's schooling are influenced by socio-economic and household demographic characteristics, including parents' education, level of wealth, level of occupation, and gender.

Basu and Tzannatos (2003) argue that for many households, child leisure or schooling is a luxury good, and if household income is not sufficient, families cannot afford to keep a child out of productive work or send them to school. Thus, child labor is often a substitute for adult labor. Diamond and Fayed (1998) discovered substitutability of adult and child labor in Egypt, and observed females are substitutes for children, but males are complements. In the case of Bangladesh, Salmon (2005) found that children and mothers are substitutes for each other as children of employed mothers were mostly working in households. Hosen et al. (2010) highlight that many poor and vulnerable parents in Bangladesh often have no alternatives to trade-off child education for paid or unpaid child work.

69.3% of rural parents engage in child labor to increase family income, 4.3% to repay loans, 3.7% for not being able to bear educational expenses, and 4.7% for children's unwillingness to study.

### Intra-Household Demand and Supply of Child Labor

It is generally agreed that higher school expenditure (fees and other expenses) may induce parents to pull their children out from school (Sabates et al., 2013). However, due to seasonal variation in agricultural activities, particularly in Bangladesh, the adults of the families may be engaged in non-agricultural work to increase income. This may create the substitution effect on the children in the household activities when the adults are busy working outside (Ahmed and Ray, 2011). Arends-Kuenning and Amin (2004) revealed that boys are mostly affected by seasonal agricultural labor demand as they are pulled out of school to meet this increased demand. This usually interrupts their school attendance and performance and thus may result into them dropping out. However, households usually tend to hire adult labor for family farming, which creates an opportunity for children to go to school. But, due to the moral hazards of managing hired adult labor, households may prefer their own family's labor as family members (especially children) are easy to supervise, and acquainted with the farm (Bhalotra and Heady, 2003). Moreover, levels of household indebtedness may have a negative correlation with child schooling. For example, if a household is in high debt, to pay it off, the adults of the households are likely to need to work longer hours which may create a necessity for children, particularly girls, to help in domestic chores (Ahmed and Ray, 2011).

## Child Labor and Schooling Situation in Bangladesh

In Bangladesh, the government provides the mainstream formal primary and secondary education for all public-school students. Primary education consists of a 5-year cycle, and secondary also has a 5-year cycle. The official age of entry into primary school is 6 years, although enrolment at later ages is also quite common. The country introduced compulsory primary education in 1992 along with free textbooks and tuition fee exemption for all children up to grade 5 and up to grade 8 for girls (Ahmed and Ray, 2011). According to Annual Primary School Census 2019, overall enrolment rate in primary school was 97.74–97.65% for girls, and 98.01% for boys. The overall student attendance rate was 88.66%; the primary education cycle completion rate for girls was 83.20%, for boys, this was 80.80%. The overall drop-out rate stood at 17.9%—for girls, it was 15.7%, and for boys, 19.20% (GoB, 2019). The COVID-19 pandemic has significantly worsened the situation (Emon et al., 2020; Hoque, 2020). An academic year of schooling has ~220 school days. Because of the government incentives (i.e., free textbooks, free tuition, free food), the parents can compensate for the foregone earnings of their children (if they had worked instead), which inspires them to send their children to school.

Bangladesh Child Labor Survey 2013 estimates that the number of total working children aged 5-17 was 3.45 million (male 2.10 million and female 1.35 million), and the number of children engaged in labor was 1.7 million (BBS, 2015). The survey defined the terms “working children” and “child labor” based on the principles of the 18th International Conference on Labor Statisticians and Bangladesh Labor Act 2006.^1^ The number of children aged between 14 and 17 engaged in child labor was 1.21 million. The national survey further informs that about 67% of child labor takes place in the rural areas, and of the children employed, 29.9% were engaged in the agricultural sector, while 33.3% were engaged in the manufacturing industry. Among rural child labor, 63% of children were not attending school during the reporting, 8.4% had never attended school, and the remaining 28.6% combined work and schooling. During the survey, households reported various reasons causing child laborers to drop out of school. As Table 1 shows, 36% of child laborers dropped schooling to support their family income, 15.1% for not being able to afford the expenses, and only 16.1% left school to start working. Notably, among three age groups (6–11, 12–13, 14–17), more than 1 in 4 children aged 6–11 dropped school to start working, and a significant of children aged 6–13 reported “no school nearby” as a reason for their dropout.

**Table 1:** Distribution of child labor by the reasons of being dropped out, by age group in Bangladesh (Source: BBS, 2015, p. 80).

**Reasons**	**6–11**	**12–13**	**14–17**	**Total**
		**%**		
Failed examination	6.1	3.4	4.5	4.7
Not interested	8.3	10.2	16.2	15.0
To start working	26.2	15.1	14.6	16.1
To get married	0.0	0.0	3.6	3.0
To support family income	10.6	13.6	16.1	15.3
Parents did not want	8.1	0.0	3.8	4.2
No school nearby	17.8	34.5	2.0	5.0
Could not afford	20.9	20.9	38.9	36
Others	2.0	2.2	0.4	0.7
Total	100	100	100	100

The report conveys Bangladesh's national definition of child labor as:

“Child labor is paid or unpaid work that is mentally, socially or morally conjugated with danger to children or the infliction of harm to children; activities that deprive children of the opportunity to go to school, or in addition to schoolwork and household responsibilities, loads additional work done in other places, which enslaves children and separates from their families; work performed by a child under the minimum age for entering into employment relationship with the employer according to the labor legislation of Bangladesh.” (BBS, 2015, p. 16)

Bangladesh has been in active collaboration with ILO-IPEC initiatives through adopting the elimination of child labor its policy documents such as “The National Child Labor Elimination Policy, 2010” focus on the planning and implementation of various short, medium and long-term strategies and programmes for withdrawing working children, particularly from the worst forms of child labor, from the workforce, and getting them out of the vicious cycle of poverty and to get them back to school (GoB, 2010). The government has enacted some laws and legal provisions in relation to ILO Convention No. 182 and UNCRC, which include the Constitutional provisions (Art. 18, 20, 34), Penal Code (Secs. 366, 372, 373, 374), Children's Act, 2013; Suppression of Violence against Women and Children Act, 2000 (Sec. 8, 9)—amended in 2003, Suppression of Immoral Traffic Act, 1933, Bangladesh Labor Act, 2006 (Chapter VII), and the Employment of Children Act, 1938 (ILO guidance). The Bangladesh Labor Act, 2006 outlaws the employment of any child <14 years of age and bans anyone below 18 in some categorized list of hazardous works (GoB, 2015). Notably, on 22 March 2022, the country, as a part of the government's commitment under the National Action Plan on the Labor Sector of Bangladesh (2021–2026), ratified ILO's Minimum Age Convention keeping 14 as the minimum age of entry to work (ILO, 2022).

## Methodology

The review of literature has illustrated various kinds of determinants of parents' decision-making on schooling or child labor, luxury and substitution axioms, intra-household demand and supply of child labor, opportunity cost of child labor and schooling, the relationship between human capital formation and child labor, and the picture of child labor and schooling in Bangladesh. However, households' decision-making patterns about child labor or schooling and the impact of parents' labor-intensive occupation in rural contexts of Bangladesh have not yet been adequately explored with qualitative insights.

### Research Design

This study aims to analyze the factors behind Bangladeshi parents' decision-making on whether to send their child to school or to work. The researchers largely depended on interpretivism (i.e., the reality is multi-layered and complex) to answer the research question. Interpretive research unpacks how people ‘feel about the world and make sense of their lives from their particular vantage points' (King et al., 2019). The qualitative research strategy has dictated the in-depth case studies and interviews which the authors conducted during their fieldwork in Bangladesh. Debout (2016) notes that a qualitative case study permits an investigation to explore various interacting factors resulting in a complex phenomenon. Such studies cognize the subjective meaning that individuals bring to their complicated and multi-layered situations (De Vaus, 2001). Understanding the attitudes, beliefs, and views of the parents regarding the trade-off between child schooling and labor necessitated the exploration of the factors responsible for their respective decision-making behaviors.

**Figure 1:**
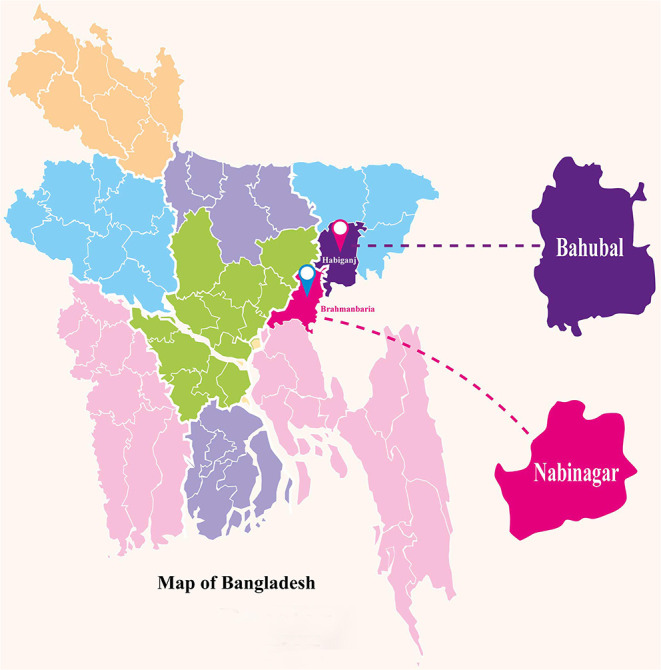
The study areas (illustration: Authors' own).

Bangladesh has a large population (about 160 million) dispersed all over the country (Tama et al., 2021). The authors intentionally chose to conduct their fieldwork in two *Upazilas* (i.e., small geographical and sub-district administrative units) named *Bahubal* and *Nabinagar* which are in the districts of *Habiganj* and *Brahmanbaria*, respectively (see Figure 1). These areas reside in the eastern part of the country. Reportedly many children in these areas are engaged in paid work in both the formal and informal sectors (GoB, 2020).

As shown in Table 2, the 2011 government estimates report that Nabinagar has 220 schools for 94,871 households, while in Bahubal138 are located for 37,334 households. Children in these areas are generally engaged in labor in small businesses, agricultural and tea farms, welding, transports and so forth. The literacy rate of Nabinagar is 68.62%, while that of Bahubal is 39.4% (GoB, 2020). The representing two districts have a significant difference in geographical features. While most of Brahmanbaria is agricultural plain land, Habiganj has hills, tea estates, haors,^2^ and forests. People in hilly regions (including Habiganj) have long suffered from regional disparities in education in the country, resulting in a low literacy rate (Khan and Islam, 2010). UNICEF Bangladesh (2020) reports the school dropout rates in the country as 5% in the 5th grade, 6% in the 8th grade and 15% in the 10th grade. These two different geographical units (Nabinagar and Bahubal Upazilas) were chosen because of their similarity in having reportedly high dropout rates (Hossain and Akter, 2019) and variation in literacy rates (see Table 2) despite being geographically close to each other, making it an interesting proposition. Hossain and Akter (2019) revealed that the reasons behind dropouts in Brahmanbaria and Habiganj districts are poverty, early marriage, and various forms of employment for children.

**Table 2:** Relevant demographics of the selected two Upazilas (GoB, 2020).

**Name of the Upazila**	**Area (km^**2**^)**	**Households**	**Schools (primary to higher secondary)**	**Literacy rate**
*Nabinagar*	350.328	94,871	220	68.62%
*Bahubal*	250.66	37,334	138	39.4%

### Sampling and Research Methods

The existing body of literature primarily informed this qualitative study. The primary data was collected from purposively and carefully selected household cases. The conditions of being considered as cases for this study are—(i) the household must have children aged 5–17; (ii) the parents must have life experience and knowledge regarding the trade-off between child labor and schooling; and (iii) the informed consent of being a participant in this research could be obtained. The initial access to the research areas was obtained through the local government offices. The Upazila administration (UA)^3^ connected the researchers with local contacts, including a few non-government workers and schoolteachers. Then, a snowball technique was employed for selecting the households and interviewees. Before selecting any household, the fulfillment of qualifying conditions was confirmed through informal discussions with its head. Two methods of data collection—semi-structured interviews and focus group discussions—were adopted to conduct the study among the selected homogenous case households and related key informants.

#### Interviews

Well-executed semi-structured interviews can provide the objectivity and trustworthiness of qualitative studies and deliver plausible results (Kallio et al., 2016; Hoque and Tama, 2021). Considering the data needs and nature of the study, one-on-one semi-structured interviews were chosen and conducted with 15 heads of households. Among these individuals, 10 were men and five were women, and three of the women were single females. The occupational status of the people interviewed were as follows: day-laborer (5), farmer (4), fisherman (3), and small entrepreneurs (3). Besides these people, four local government officials and two officials of local non-government organizations (NGO) having relevant work experience were also interviewed. These key informants were all male. Due to unavailability, it was not possible to ensure female representation. However, these additional interviews provided this study with complementary data, which were helpful to build the whole picture. Each interview lasted about 40–60 min; however, additional time was required to reach the households due to underdeveloped rural communication.

#### Focus Group Discussion

In qualitative research work, Focus Group Discussions (FGD) are considered effective tools for stimulating opinion, engaged debates, and in-depth rationale of actions and behaviors. It is conducted with a purposefully selected group of individuals to gain an in-depth understanding of social issues (Woodring et al., 2006; Nyumba et al., 2018). In such discussions, participants are normally naturalistic and tend to re-evaluate their existing position (King et al., 2019). To cross-check the validity of interview data, and to explore additional parental views, two FGDs were conducted with the parents of the affected children in the two selected Upazilas. Each group discussion included participation by six parents—four males and two females. The FGDs were conducted after completing all individual interviews. The interviewees (schoolteachers, parents and local education officers) assisted the researchers in finding suitable participants for FGDs. These FGDs were carried out in November 2020 when government restrictions on public movement and activities were already lifted (Adhikary and Habib, 2020). However, safety measures were maintained while conducting these sessions. Participants only from close distances could join the discussion. Each discussion lasts about 2 h. The topics were guided by a set of open-ended questions which allowed the participants to dig deeper into the issues and provide useful insights.

### Data Analysis, Ethics and Limitations

After completing the 4-week period of data collection in October–November 2020, data were transcribed, compared, and analyzed with regards to the existing body of knowledge and the research questions. Similarities with existing theories and empirics were noted down. A narrative style of data analysis and interpretation was employed to understand the reasoning of behaviors, attitudes, and beliefs of the participants. Narrative analysis is centered on the assumption that people use stories to make sense of themselves, and deals with how the narrator justifies or makes sense of the interpretations (Frost et al., 2010). This analysis was helpful to explore the factors of making a particular trade-off decision, and to report real scenarios. The aim was to understand the interviewees' views, perspectives, and beliefs and generate deep insights, rich descriptions, and engaging exploration. Focusing on an attainable number of individual interviews and household cases allowed the analysis to realize that direction. The analysis provokes thoughts and provides directions for future research.

Ethical standards of interviewing were strictly followed while collecting data for this study. Interviews were conducted ensuring that the local COVID-19 pandemic related health guidelines were maintained, and no psychological harm occurred. Informed consents were obtained in all cases. However, the main limitation of this data and research is that the fieldwork is based in a specific geographic area, time, and context. Readers must be aware that the findings are drawn from a relatively small number of interviews and cannot be generalized to a broader context. Neither FGDs nor the interviews are representational. These findings should neither be used to compare the two Upazilas' child labor/schooling situations. In addition, although the data collection was carried out carefully, there might be social desirability bias to it. Some data may reflect mere opinion of the participants rather than their real-life experiences. As most of the parents interviewed were illiterate, the role of parental education could not be well-examined.

## Findings and Analysis

The primary research and secondary review guided the examination of factors affecting parents' decision making on child's schooling or having them work. The assessment emphasized on testing some of the common issues regarding child labor and schooling in rural areas. The empirical analysis of this study is based upon the individual interviews and FGDs in rural Bangladesh. The following sections delineate the key findings and analyses.

### Poverty Remains the Most Cited Factor

Parents, among all factors, mainly highlight their economic impoverishment for trading off child labor with schooling. Almost all the parents emphasized that poverty is the main reason behind their decision on not sending children to or pulling them out from school, and/or engaging in child labor. As one respondent stated:

I have my 14-year-old son, who has studied up to class (grade) ten. He has been absent from school for one year. Now he is working in Dhaka. Due to the financial crisis, I had to stop my son's education. I had to take the decision since I could not bear the extra expenses on top of the regular fees. (*Interviewee-10*)

Shafiq (2007) noted that the average annual educational costs at primary, junior-secondary, and higher-secondary levels in Bangladesh were approximately Bangladeshi Taka (BDT) 517 (~$ 6),^4^ BDT 2515 (~$ 30), and BDT 4559 (~$ 53), respectively. Even including the inflations over the years, this expenditure remains considerably low. Such low expenditure may not seem to be substantial enough for stopping schooling. However, for the marginal poor households this might be challenging to bear. Furthermore, the opportunity of added income by the children may induce them to think of trading off schooling with child labor. Another respondent shared that his income is not enough to take care of his family, and he has been compelled to engage his children into labor instead of sending them to school, which is consistent with Basu and Van's (1998) luxury axiom that if the total income of a family is below the subsistence level, the children of the family are compelled to get involved in labor. Debt is also a major driver for child labor for many households. Another respondent shared:

I have some loans. As my sons work with me, it has become easy for me to continue the installments of those loans. (*Interviewee-2*)

In some cases, children are also used as substitutes for adult laborers in family businesses where parents directly employ them. Thus, children can often be the last resort of economic resource.

### Single-Headed Households Tend to Engage Child Labor

Single parent headed households are often compelled to trade off schooling with child labor. The female-headed households, particularly, cannot bear the educational expenses or the opportunity cost of foregone income by the children. Such household-heads share that their primary concern is to ensure food and shelter for their family. A widow shared her helplessness regarding her son's schooling:

My husband died several years ago. I do not have a regular job. I found it impossible to continue my son's schooling. A local barber shop offered him a job and now, he earns too. (*Interviewee-8*)

Another female household head described similar conditions which echoes Goldin's (1979) observations for the urban labor force participation of children in the United States. However, this finding contradicts Binder and Scrogin's (1999) hypothesis that children in female-headed household have a lower chance of having to work. If the main breadwinner (the male adult in most cases) dies, the mother of the family has to struggle to manage subsistence level survival for her family. To increase the total income of the household, the household seeks for other members' support, regardless of whether they are adults or children. In such circumstances, they generally depend on the older (aged 12–17) children and, ultimately trade-off schooling with child labor.

### Taking Care of Younger Siblings Is Crucial

Shedding light on why some older children do not complete school, one Upazila Education Officer (UEO) pointed out that older children often have to take care of their younger siblings at home. Households particularly dependent on agricultural farming are generally engaged with various farming-related activities. Often both parents remain busy all day with farmstead and homestead works. As a result, older children give care to younger ones in households with a comparatively large number of members. Salmon (2005) also identified the number of household members as a major factor in Bangladesh when it comes to children being engaged in labor. However, some parents argued that they do not want to stop their children's schooling, but that rather it is the children themselves who do not like to go to school for various reasons. For example, one father stated:

My elder son is 16. He completed up to grade 6. He was weak in studies. He could not bear the pressure of studies in his school. He used to often get sick. So, he has stopped going to school. We (both parents) want him to go to school, but he says he does not like school. (*Interviewee-1*)

This statement indicates that the schools fail to attract children for various reasons. The COVID-19 pandemic has made the situation worse. The long closure of in-person schooling has pushed some children to drop out of education in Bangladesh forever.

### Parental and Cultural Beliefs About Children's Roles Are Important

Most parents who were interviewed expressed their dislike of the leisure enjoyed by boys. They think boys should always be doing something productive. If any school-going child does not go to school or drops out, parents do not allow them to go idle. This tendency has been obvious during the COVID-19 pandemic. The long closure induced many poor households to send their children, particularly boys, to labor. As one respondent acknowledged:

Due to the COVID-19 pandemic, the school has been closed. My son did not have to go to school anymore. We don't have the (digital) means to let him do the classes online. Meanwhile, he got an opportunity to work as an apprentice in the local market. (*Interviewee-7*)

Sexual division of labor is obvious as most girls were seen working inside homes while boys work outside. Several parents believed that homestead jobs should be performed by the girls, while boys should work outside in the marketplace or family farms to develop their skills. Availability of paid jobs allocated for boys, and unpaid jobs for girls is also responsible for this sexual division of labor.

The findings of this study indicates that boys are mostly pulled out from the school rather than the girls. One father of a boy and a girl studying in the same grade (7th grade) in the same school, shared that due to the growing expenditure he had to pull his son out of school and engage him in labor work. Another respondent who has two daughters and one son, kept sending the girls to school but did not allow the boy to continue. An NGO worker explained this trend:

Nowadays parents are not interested to send their sons to school. But in case of their daughters, they are interested to some extent. Because they believe boys can earn money even without being formally educated. (*Interviewee-22*)

Probing this observation further reveals that a lot has to do with the government incentives provided for school going students. Parents find it beneficial to keep girls (as opposed to boys) enrolled through secondary schooling as they receive incentives from the government. More importantly, girls can combine homestead unpaid work with schooling. One Higher Secondary Education Officer (HSEO) reflected:

Girls receive regular stipends from schools. While making a trade-off decision, parents tend to send sons to paid works, and ask their daughters to continue with schooling to receive government incentives. (*Interviewee-23*)

Therefore, to the parents, the opportunity cost of sending boys to school is greater than for girls, which is similar to the results that (Arends-Kuenning and Amin, 2004) found in their study on the impacts of incentive programmes on children's activities in Bangladesh. Traditionally, patriarchal norms and beliefs remained obstacles to girls' advancement and development in rural areas (Parveen, 2007; Islam and Sharma, 2021). Yet, the country has made tremendous progress in girls' education in rural areas (Tanaka et al., 2021). However, strong cultural beliefs are critically unfavorable for girls' education, including a religious convention mentioned by a respondent in FGD:

There is a convention in Muslim community that girls do not require higher education. If they can complete primary education, then it is good enough to marry them off. (*FGD-1, Bahubal*)

Emerging evidence shows that education institutions' extended closure (nearly 2 years) has had severe negative impacts on child marriages and dropouts in Bangladesh (Hussain, 2021). A recent report reveals that the number of child marriages in Bangladesh has increased by 13% during the pandemic (Dhaka Tribune, 2021). The pandemic also led to a sharp rise in child labor (Financial Express, 2021). Moreover, parents usually send their daughters to school for immediate returns, instead of an ambition of any long-term return; they want to marry them off as soon as possible. Hence, in most cases, girls' education stops at the end of the secondary school level. One parent with the same mindset shared:

I wish, my daughter successfully passes secondary school. Then it will be easy for us to arrange a good marriage for her. *(Interviewee-9)*

Interviews with parents of full-time working and non-school going children reveal that such parents are mostly illiterate. It indicates that parents' illiteracy may have negative impacts on their children's schooling and a positive relationship with child labor incidences (i.e., the less literate the parents, the more likely that their children are engaged in child labor).

### Educational Expenditure and Structural Factors Related to Schooling Are Critical

In Bangladesh, primary-level schooling is free from tuition fees for everyone, while at the higher secondary level, schooling is free for girls only. The government provides books for free as well. However, parents must bear some extra educational expenses for buying additional books and materials. Some households also need to pay for the travels to school. Poor households find it hard to bear these expenses. The following statements clearly reflect this issue.

I cannot afford to pay for both of my children; apart from fees, running expenses for schooling are too high for me (*Interviewee-10*).

For educational materials government can give us allowance because we are destitute and helpless. (*FGD-2, Nabinagar*)

High school expenditure may induce parents with low incomes to pull their children out of school (Sabates et al., 2013). In Bangladesh, there is no such credit available for the poor with which they can maintain their children's educational expenditure. Though the government provides incentives for school-going girls, parents believe this support is not enough. In rural areas where government-run schools are far from home, parents sometimes send their children to non-government schools. However, as indicated in one of the above statements, non-government schools are relatively more expensive. An FGD participant raised this issue:

The government-run school is too far to walk to. Our children attend a nearby private school, and the cost is very high. (*FGD-2, Nabinagar*)

Lack of availability and poor facilities of government-run schools also work as a crucial deterrent that drive children away from attending these schools. Huisman and Smits (2009) also noted similar observations regarding educational facilities for rural children in developing countries. One participant in FGD elaborately stated:

In this remote village, I cannot ensure a fitting environment for my children. Teachers in school do not give them adequate time and care. If I could manage a good house tutor, they would do much better in their studies. My son was in grade four, but he left school a couple of years ago. Now, he does nothing – neither study nor work. I hope he can do some paid jobs to acquire skills in the coming years. (FGD-1, *Bahubal*)

Parents value the skill development of their children since skills have immediate demands and returns through paid jobs. On the other hand, formal education is a long-term investment that parents do not find worth the money. In addition, schooling is not always considered an investment in human capital. When parents think their children are not receiving quality education or not doing well enough, they tend to start thinking negatively about investing in human capital. One UEO stated that parents often believe that learning skills though working is more valuable than education.

### Intra-Household Demand and Supply of Labor Happens

During the season of peak agricultural activities, the adults of the households (both male and female) get engaged into farmstead and homestead works. Girls are additionally employed in household work to help their mothers and boys are employed in the field to help their fathers. Generally, poor families cannot hire people to meet this higher demand for labor, so instead, they pull their children out of school. An NGO officer said that when the demand for labor increases, households use children as laborers to save on costs. One female parent in the FGD stated:

During the harvesting season, we cannot send our children to school. We need their help to fill the labor shortage. They help to save expenses. (FGD-1, *Bahubal*)

Therefore, irregular attendance and drop-outs are normal during the harvesting season. The participants informed that the decision of using children in family farming is usually taken by both parents (if available) together. The following statement from one HSEO further illustrates this issue.

There is a negative impact of the harvesting season on children's school attendance. In the rural areas, most poor households withdraw their children from schools and engage them in harvesting work. It has become almost a culture here. (*Interviewee-23*)

### Parents Engaged in Labor-Intensive Occupations Tend to Trade-Off More Frequently

When non-parent respondents were asked to mention the type of occupations people were employed in which generally led to those households' children being more likely to be engaged in child labor, they mentioned low-income households headed by farmers, day-laborers, tea garden workers, fishermen, and rickshaw pullers. All the respondents of this study who engaged their children into labor are in low-income occupations. From our study, one male parent who runs a construction business stated:

My elder son completed secondary school and works in construction with me. My second son studied up to class six and now works with me. My third son is in class four, and he is too young to help me in my business or works. (*Interviewee-2*)

Another father engaged in small business said that one of his sons was engaged in his shop instead of going to school which also strengthen the hypothesis that if parents need labor, they tend to engage their own children. One HSEO corroborated this by saying that low-earning parents generally engage their children in their own occupations. The six officials interviewed in this study affirmed that the rate of school dropouts from households headed by labor-intensive occupations is higher than others. One respondent in an FGD stated:

I have my own shop. My sons help me. It helps us to earn more profit since I do not have to hire labor. This way, they can also learn how to run businesses. (FGD-2, *Nabinagar*)

This indicates that this tendency may not be merely driven by subsistence needs, and altruistic parents often want their children to follow their footsteps as well.

### Which Interventions at the Government and Non-government Levels Can Be Effective?

The respondents were asked what kind of government or non-government organizational support would help them to keep their children in schools. Many respondents mentioned credit facilities and cash and in-kind transfer support. Lack of credit facilities are associated with some of the crucial factors affecting child schooling and encouraging child labor engagement instead. If the adult members of the household can create any opportunity of sufficient earning with credit facilities, it can reduce subsistence challenges. Parents note that suitable credit facilities could help them to improve their marginal income. It would then be easy for them to send their children to school or carry on with their schooling. How credit facilities can increase opportunities for sending children to school has been described by one of the FGD participants:

If parents can earn by investing in a small business, it will generate additional financial resources. Then, they will not be forced to utilize the labor of their children. Big families can also afford to send their children to schools. (*FGD-2, Nabinagar*)

Sometimes parents want their children to combine schooling and household work. They think that a little help from their children in homestead work will not hamper their children's studies. Credit-run businesses can allow more parents to do so. As one respondent stated:

If I could get loans with which I could buy livestock like cows or goats, then I could send my son to school. The return from the livestock would be helpful. All our family members can work together to take care of the livestock. (*Interviewee-4*)

One NGO worker believes that if households had access to credit, they would not withdraw children from schools even in the harvesting season. They could instead afford to hire adult laborers. However, some of the respondents mentioned some procedural disadvantages regarding credit receiving and repayment. A substantial number of respondents shared their fear of managing installment repayments. To pay the installment every week or month sometimes becomes troublesome for them. They said that they worried they would not be able to make profit due to investment imperfections like failure to invest in proper fields, and consequently fall into unbearable liabilities. Some of them do not want to receive any more loans. Furthermore, the high rate of interests, especially from the NGOs, is also a concern for them. As one respondent stated:

Some of us are afraid of getting any loans because it becomes hard for us to pay installments regularly. We have not applied for any loans in the recent past. (*Interviewee-1*).

The issue of debt remains very crucial for many households. Also, some households who received loans against their agricultural farms from NGOs could not invest in their intended purposes. For example, one household spent their entire loan amount on building a house as she had no house to live in. Another respondent said that he has spent some of his loan in buying medicines for his family. Both families depend on child labor to some extent. They think if the installment and the rate of interest would have been favorable, they could have benefitted from the loan to a greater extent. As one respondent stated:

Paying weekly installments sometimes becomes hard for me. If it could be paid after three or four months, then it would be much easier. In addition, the rate of interest is also too high for us. (*Interviewee-2*)

Some parents believe that conventional credit facilities are not suitable for poor households. Generally, poor households receive an amount of credit which is too little to start any profitable enterprise. They think interest-free loans or government allowances would work better in terms of helping them to make their children free to go to school instead of having to work. As one FGD participant shared:

We are afraid of loans because of their high interest rates. We need interest-free loans worth BDT. 50,000 to 100,000. Besides, government can give allowance per child of around BDT. 3,000 to 5,000 per month so that we can send them to school. We need support for our boys too. (FGD-2, *Nabinagar*)

However, they did not dig into the phenomenon that most parents having small businesses also tend to use child labor—either from their own family or from other sources. Small businesses tend to employ children to save money. Evidence suggests that transfer programs are generally more effective in reducing child labor (Dammert et al., 2018). Nevertheless, there needs to be policies that protect and support such poor households.

## Conclusions

This study explored the factors behind the trade-off between children's schooling and labor. The empirical analysis also identified some crucial determinants of the issue. The results confirm earlier findings that households which are at below subsistence levels of income are more likely to trade off their child's schooling with child labor. Though the primary and secondary school expenditure in Bangladesh is relatively low, even then, the marginal poor households do struggle with the costs. Parents depend on their children's economic activities to both generate income and reduce household expenditure. Furthermore, studies also show that single parent female-headed households tend to trade off schooling with child labor to a greater extent than other household groups. In poor households, older children often have to take care of younger siblings, which affects their school attendance. Due to the sex-disaggregated division of labor, boys are mostly engaged in market work and girls in household work. Moreover, girls are sometimes discriminated against in terms of getting a proper education or being engaged in more productive work because of social and cultural beliefs. From a patriarchic social point of view, many people in rural Bangladesh believe that girls do not need higher education as they should not work outside the home. Because of this, many poor and ignorant parents tend to stop their girls' education quite early. There is also a pervading cultural belief that children should not go idle. The combination of these social and cultural beliefs mean that parents often encourage their children (both boys and girls) to work instead of going to school. Boys are more likely to be pulled out of school rather than girls since boys have more demand for work in paid jobs (e.g., workshops, saloons, agricultural farms) away from home. Boys also do not receive adequate government allowances (compared to girls). Furthermore, parents' lack of education has a strong correlation with trading-off schooling with child labor.

In addition to these factors, the lack of adequate educational facilities, additional schooling expenses, and poor quality of teaching also influence parental decisions in terms of whether to send their children to school or not. The empirical findings from this study indicate that parental expectation on the importance of their children's education is strongly associated with children dropping out of school and practicing child labor. This means that if parents put a low value on their children's education, the more likely they are to drop out and get engaged in child labor. The findings of the study suggest that sometimes households view children as the last resort to meet their economic or labor needs. Parents employed in labor-intensive occupations are also more likely to engage their children into labor work instead of sending them to school.

Finally, the findings also suggest that parents have mixed views regarding credits and loans. Some are in favor of credit as a means of business support, while others fear it for its high interest rates. Analysis highlights that support programmes, especially cash and in-kind transfers, influence parental attitudes and behavior concerning this trade-off. However, bringing positive outcomes toward increasing child schooling would require carefully designed interventions. Since parents' decision-making on the issue does not merely depend on their household characteristics but rather on various kinds of socio-economic and cultural factors, support interventions must target the underlying causes of child labor.

## Data Availability Statement

The raw data supporting the conclusions of this article will be made available by the authors upon reasonable request.

## Ethics Statement

The risk assessment and ethics of the field research of this study were reviewed and approved by the University of Birmingham, UK. The participants provided their written informed consent to participate in this study.

## Author Contributions

Both authors have made a substantial, direct, and intellectual contribution to the work and approved it for publication.

## Conflict of Interest

The authors declare that the research was conducted in the absence of any commercial or financial relationships that could be construed as a potential conflict of interest.

## Publisher's Note

All claims expressed in this article are solely those of the authors and do not necessarily represent those of their affiliated organizations, or those of the publisher, the editors and the reviewers. Any product that may be evaluated in this article, or claim that may be made by its manufacturer, is not guaranteed or endorsed by the publisher.

## Footnotes

^1^Bangladesh Labour Act 2006 defines a child as a person who has not completed his fourteenth year of age. However, the National Child Labour Survey considered the age group of 5–17 as children.

^2^A Haor is a bowl-shaped large tectonic depression that receives water from rivers and canals to become an extensive water body during monsoon. For more details, visit https://en.banglapedia.org/index.php/Haor (accessed on 16 April 2022).

^3^UA as a government administrative body represents the national government at the Upazila level. The country has 492 Upazilas (Mahmud, 2021).

^4^1 United States Dollar values ~86 Bangladeshi Taka. Source: https://www.xe.com/currencyconverter/convert/?Amount=1&From=USD&To=BDT (accessed on 5 December 2021).
